# An ecological assessment of the potential pandemic threat of Dengue Virus in Zhejiang province of China

**DOI:** 10.1186/s12879-023-08444-0

**Published:** 2023-07-17

**Authors:** Yaxing Zhang, Lei Wang, Guozhen Wang, Jiabao Xu, Tianxing Zhang

**Affiliations:** 1 College of Basic Medicine, Zhejiang Chinese Medical University, Hangzhou, 310053 China; 2 Clinical Practice Teaching Center, Academic Affairs Office, Zhejiang Chinese Medical University, Hangzhou, China

**Keywords:** Dengue virus, MaxEnt, *Aedes albopictus*, Climate change, Jackknife test

## Abstract

**Background and Aim:**

Dengue fever, transmitted by *Aedes* mosquitoes, is a significant public health concern in tropical and subtropical regions. With the end of the COVID-19 pandemic and the reopening of the borders, dengue fever remains a threat to mainland China, Zhejiang province of China is facing a huge risk of importing the dengue virus. This study aims to analyze and predict the current and future potential risk regions for *Aedes* vectors distribution and dengue prevalence in Zhejiang province of China.

**Method:**

We collected occurrence records of DENV and DENV vectors globally from 2010 to 2022, along with historical and future climate data and human population density data. In order to predict the probability of DENV distribution in Zhejiang province of China under future conditions, the ecological niche of *Ae. aegypti* and *Ae. albopictus* was first performed with historical climate data based on MaxEnt. Then, predicted results along with a set of bioclimatic variables, elevation and human population density were included in MaxEnt model to analyze the risk region of DENV in Zhejiang province. Finally, the established model was utilized to predict the spatial pattern of DENV risk in the current and future scenarios in Zhejiang province of China.

**Results:**

Our findings indicated that approximately 89.2% (90,805.6 KM^2^) of Zhejiang province of China is under risk, within about 8.0% (8,144 KM^2^) classified as high risk area for DENV prevalence. *Ae. albopictus* were identified as the primary factor influencing the distribution of DENV. Future predictions suggest that sustainable and “green” development pathways may increase the risk of DENV prevalence in Zhejiang province of China. Conversely, Fossil-fueled development pathways may reduce the risk due to the unsuitable environment for vectors.

**Conclusions:**

The implications of this research highlight the need for effective vector control measures, community engagement, health education, and environmental initiatives to mitigate the potential spread of dengue fever in high-risk regions of Zhejiang province of China.

**Supplementary Information:**

The online version contains supplementary material available at 10.1186/s12879-023-08444-0.

## Introduction

Dengue fever (DF), caused by Dengue virus (DENV) [1, 2], is prevalent in tropical and subtropical regions worldwide, particularly in urban and semi-urban areas. It has become a global public health concern [3]. Severe cases of DENV can lead to complications such as plasma leakage, fluid buildup, respiratory difficulties, severe bleeding, or organ damage [4, 5]. The transmission of DENV occurs through *Aedes mosquitoes*, with *Aedes aegypti* and *Aedes albopictus* being the primary vectors in urban cycles of DENV transmission [6]. Initially identified in only nine countries in 1970, DENV has rapidly expanded to over 100 countries in the last decade due to socioeconomic changes and global climate change [7].

Dengue fever occurs throughout mainland China except in Tibet Autonomous Region [8]. From 2005 to 2020, DF cases significantly increased, with 12,701 imported cases and 81,653 indigenous cases [8]. DENV outbreaks have spread from southern coastal areas such as Guangdong [9] and Hainan [10] to more northern and western regions, including Fujian [11], Zhejiang [12], Hunan [13], Jiangxi [14], Guangxi Zhuang Autonomous Region [15], and Yunnan [16]. Recent data indicated that indigenous cases were mainly found in Guangdong (74.0%) and Yunnan provinces (13.7%) which face a continuous risk of DENV importation [8]. Zhejiang province, located in the southeast of China, falls within the subtropical zone with diverse landforms and a humid monsoon climate. *Ae. albopictus* is widely distributed in the urban and rural residential areas of Zhejiang, with high density in summer and autumn [17]. These conditions contribute to DENV vector living and DENV transmission. From 2015 to 2018, there were 1,584 reported cases in Zhejiang province of China [18]. The imported and indigenous dengue cases declined in 2020–2022 due to COVID-19 lockdown measures [8]. However, with the reopening of borders, dengue continues to pose a threat to mainland China. Thus, there is an urgent need to accesses the risk of DENV prevalence in Zhejiang province and implement enhanced public health measures against dengue fever.

The maximum entropy (MaxEnt) model is extensively utilized for evaluating and predicting the habitat distribution of species due to its inherent stability and high level of accuracy [19]. Species distribution models require the use of ecologically relevant predictor variables specific to the species under investigation. MaxEnt has found widespread application in biodiversity conservation and modeling of invasive species [20–22]. Moreover, it has been extensively employed in the context of infectious diseases [23–25] and prediction of disease vectors [26, 27]. However, researchers have used MaxEnt to model a variety of viruses including the Zika virus [23], influenza virus [28], and West Nile virus [24], no prior study has focused on spatially predicting potential DENV prevalence area in Zhejiang province of China. DENV prevalence is intricately linked to the interactions between hosts, vectors, viruses, and bioclimatic factors. Hence, this study aims to evaluate the relative importance of environmental variables and *Aedes* vectors in predicting DENV prevalence in Zhejiang province under the current conditions, as well as the potential prevalence under different developmental trajectories and future climate models.

## Methods

### Species occurrence records of *ae. Aegypti* and *ae. Albopictus*, and occurrence records of DENV

The occurrence records of DENV vectors, specifically *Ae. aegypti* (15,402 records) and *Ae. albopictus* (40,193 records), were obtained from the Global Biodiversity Information Facility (GBIF) (https://www.gbif.org, accessed on 15 August 2022) [29, 30]. Additionally, a total of 39,806 DENV occurrence records from the period between 2010 and 2022 were collected from HealthMap (https://healthmap.org/) [31]. Prior to modeling, records with inaccurate geographic information were carefully filtered out.

### Environmental variable screening and data processing

The future projection risk was estimated by using the MaxEnt model to predict the probability of DENV distribution under future conditions. First, the ecological niche of *Ae. aegypti* and *Ae. albopictus* was first performed with historical climate data based on MaxEent. Then, predicted results along with a set of bioclimatic variables were included in MaxEnt model to analyze the risk region of DENV in Zhejiang province. Finally, the established model was utilized to predict spatial pattern of DENV risk in the different current and future scenarios in Zhejiang province.

Historical climate data (2.5 min of spatial resolution) and future climate data (2.5 min of spatial resolution predicted by various climate models including BCC-ACCESS-CM2, CMCC-ESM2, EC-Earth3-Veg, GISS-E2-1-G, INM-CM4-8, MIROC6, CNRM-CM6-1, and CNRM-ESM2-1) of 19 bioclimatic variables were obtained from WorldClim (https://www.worldclim.org/, released in January 2020) [32]. Elevation data was derived from the SRTM elevation data as well as downloaded from WorldClim.

The shared socioeconomic pathways (SSPs) have been specifically designed to encompass a diverse range of potential future scenarios, effectively capturing the varied challenges and opportunities associated with mitigating and adapting to climate change. These pathways serve as valuable tools for examining the potential impacts that socio-economic trajectories can have on climate change, thereby enabling a comprehensive analysis of their implications for policy-making and decision-making processes. To ensure robustness in our study, we obtained human population density data and future projections based on the SSPs from the Socioeconomic Data and Applications Center (SEDAC, https://sedac.ciesin.columbia.edu/) [33]. The datasets acquired included historical human population density data as well as future projections, utilizing a one-eighth global degree population-based year and projection grids based on the SSPs.

All variables were resampled to 2.5 min resolution using R software (version 4.0.0). To reduce multicollinearity and minimize model overfitting, the variance inflation factors (VIFs) between variables used in the modeling of *Ae. aegypti, Ae. albopictus* and DENV were calculated, respectively. If VIF values exceed ten, the corresponding variables will be excluded. For *Ae. aegypti* and *Ae. albopictus* prediction, a total of 10 bioclimatic variables and elevation were used. These 10 bioclimatic variables were Bio_2 (Mean diurnal range), Bio_3 (Isothermality), Bio_4 (Temperature seasonality), Bio_8 (Mean temperature of wettest quarter), Bio_9 (Mean temperature of driest quarter), Bio_13 (Precipitation of wettest month), Bio_14 (Precipitation of driest month), Bio_15 (Precipitation seasonality), Bio_18 (Precipitation of warmest quarter) and Bio_19 (Precipitation of coldest quarter). In the case of DENV prediction, the anticipated distribution of *Ae. aegypti* and *Ae. albopictus*, along with the same set of 9 bioclimatic variables, elevation data, and human population data, were utilized. These 9 bioclimatic variables included Bio_2 (Mean diurnal range), Bio_3 (Isothermality), Bio_8 (Mean temperature of wettest quarter), Bio_9 (Mean temperature of driest quarter), Bio_13 (Precipitation of wettest month), Bio_14 (Precipitation of driest month), Bio_15 (Precipitation seasonality), Bio_18 (Precipitation of warmest quarter) and Bio_19 (Precipitation of coldest quarter).

In addition, in the future periods (2021–2040, 2041–2060, 2061–2080, and 2081–2100) DENV potential prevalence in Zhejiang province were also predicted under four different shared socioeconomic pathway scenarios (SSP126, SSP245, SSP370, and SSP585) of 8 climate models (BCC-ACCESS-CM2, CMCC-ESM2, EC-Earth3-Veg, GISS-E2-1-G, INM-CM4-8, MIROC6, CNRM-CM6-1, and CNRM-ESM2-1).

### Species distribution modeling of *ae. Aegypti*, *ae. Albopictus* and DENV

MaxEnt was employed to predict the distribution of *Ae. aegypti*, *Ae. albopictus* and DENV with 100 bootstrap replicates. 85% of the occurrence records were used as the training sample set, and the remaining 15% of the occurrence records were used as the test data set by using the ‘dismo’ package [34] and ‘raster’ [35] package within R software (version 4.0.0). Determined empirically regularization parameters were employed to control the model overfitting. In addition, MaxEnt generates suitable index estimating the risk of DENV. Finally, MaxEnt generates response curves and a jackknife test for individual predictors. The performance of the MaxEnt model was measured by the receiver operating characteristic curve and the area under the ROC curve (AUC). The constructed model was then used for the future forecast. To enhance the assessment of model accuracy and precision [36], we employed the maximum True Skill Statistics (TSS = sensitivity + specificity – 1) [37]. The TSS scale ranges from − 1 to 1, with − 1 to 0.4 indicating poor performance, 0.4 to 0.5 representing fair performance, 0.5 to 0.7 indicating good performance, 0.7 to 0.85 denoting very good performance, 0.85 to 0.9 signifying excellent performance, and 0.9 to 1 reflecting nearly perfect to perfect performance.

The risk area of DENV in Zhejiang province was built by the ArcGIS 10.0 software. the risk index of the risk area of the DENV epidemic was divided into four intervals by the manual grading method. High risk area: suitable index [0.60, 1.00]; moderate risk area: suitable index [0.40, 0.60); low risk area: suitable index [0.2, 0.40); no risk area: suitable index [0, 0.2). Potential total area is defined as the sum of high risk area, moderate risk area and low risk area.

## Results

### Model evaluation

**Fig. 1:**
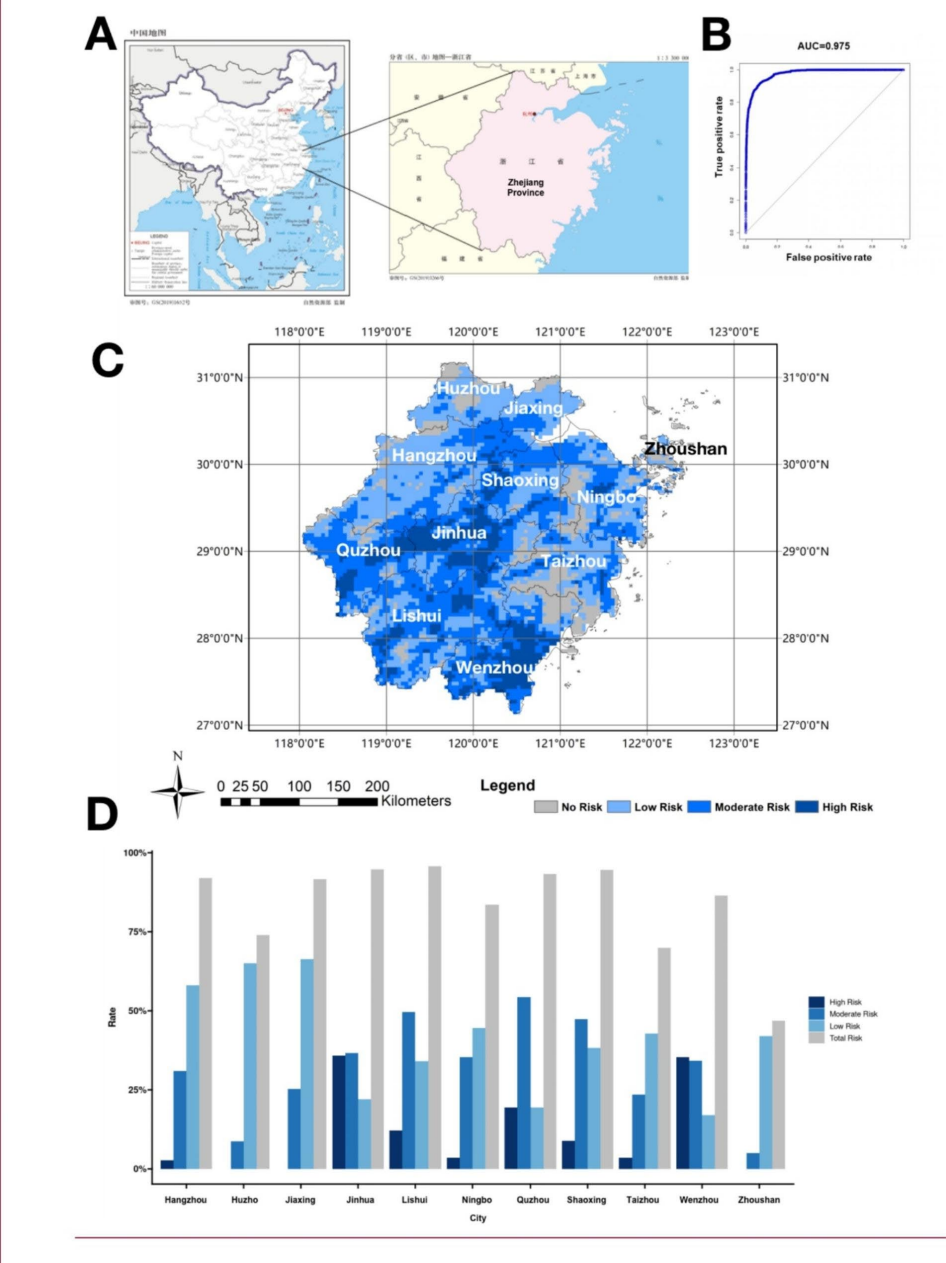
Probability of risk area predicted by MaxEnt in Zhejiang province. (**A**) The location of Zhejiang province in China. (**B**) The ROC curve and AUC value evaluating the model based on MaxEnt. (**C** and **D**) The rate of DENV prevalence risk region of cities in Zhejiang province. Values close to dark blue indicate high risk for DENV epidemic

*Ae. aegypti* and *Ae. albopictus* are the major vectors in DENV urban cycle, we first predicted the ecological niche of *Ae. albopictus* (supplementary Fig. S1) and *Ae. aegypti* (supplementary Fig. S2) by MaxEnt model with historical climate data. The training AUC for 100 replicate runs of the MaxEnt model was 0.918 (*Ae. aegypti*) and 0.920 (*Ae. albopictus*). The maximum TSS for 100 replicate runs of the MaxEnt model was 0.661 (*Ae. aegypti*) and 0.675 (*Ae. albopictus*). The risk regain of DENV of Zhejiang province of China (Fig. 1A) under current climate were analyzed using MaxEnt with bootstrap of 100. Secondly, the DENV prevalence in Zhejiang was performed using MaxEnt with bioclimatic factors, elevation, human population, and previous *Ae. albopictus* and *Ae. aegypti* results. The results showed the mean AUC value for the training set was 0.975, which indicated good model performance level and accuracy in predicting the potential DENV prevalence in Zhejiang (Fig. 1B). And the maximum TSS showed a good performance of prediction for the MaxEnt model with the value of 0.80. According to the results, about 89.2% (90,805.6 KM^2^) area in total is under risk for DENV prevalence including 8.0% area (8,144 KM^2^) with high risk, 36.1% (36,749.8 KM^2^) with moderate prevalence, and 45.1% area (45,911.8 KM^2^) with low risk. The high risk area for DENV were mainly distributed in the city of Wenzhou, Jinhua, Quzhou and Lishui which located in the south, west, west-east and middle of Zhejiang province, respectively (Fig. 1C to D). Potential suitable *Ae. albopictus* distribution area in Zhejiang province, obtained from MaxEnt are shown in supplementary (Fig. S3).

### Variable importance

**Fig. 2:**
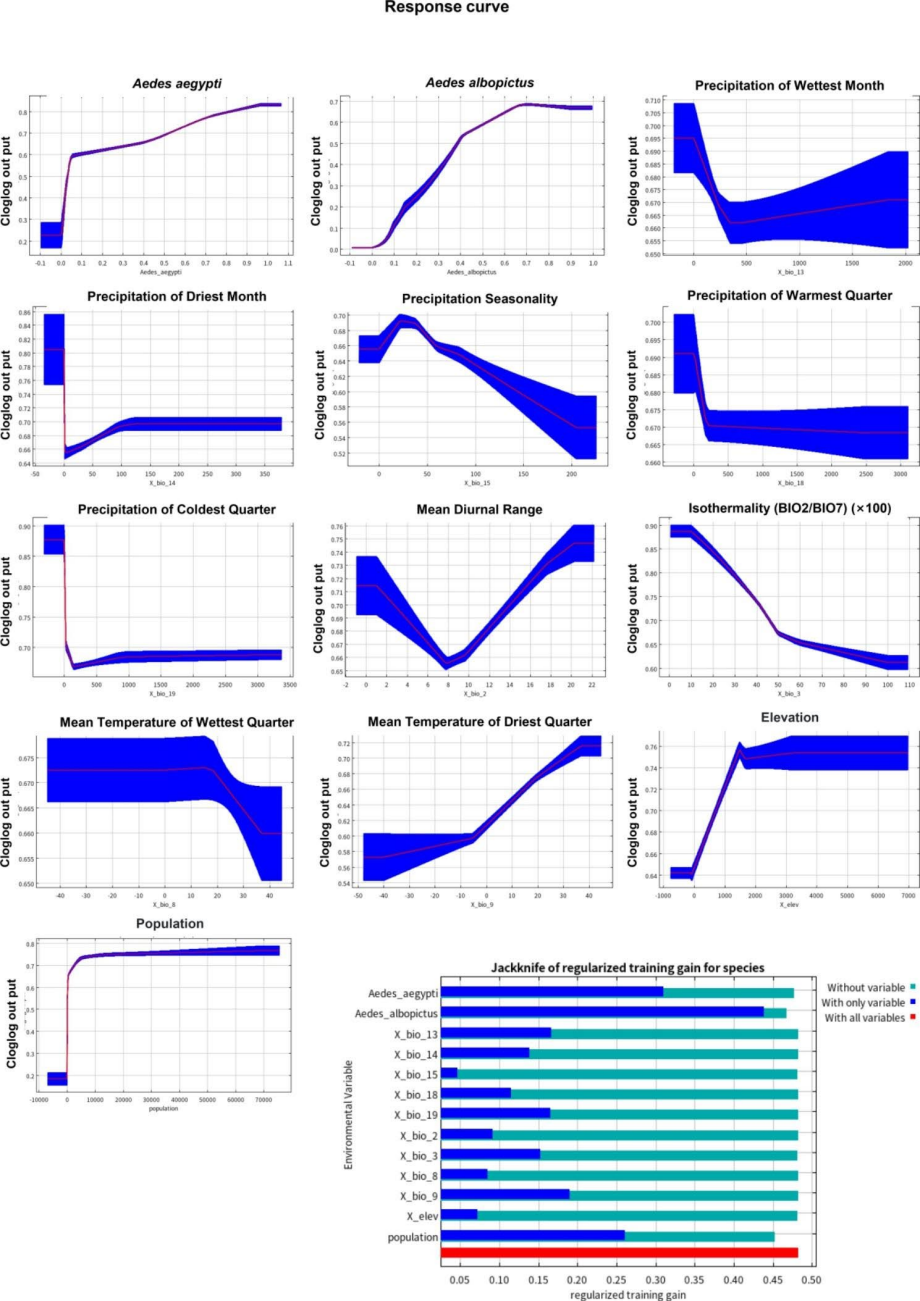
Response curve of 13 variables in DENV prevalence prediction and Jackknife test gain for DENV prevalence. Blue, green, and red bars indicated the variable alone, without the variable, and with all variables, respectively. X_bio_2, X_bio_3, X_bio_8, X_bio_9, X_bio_13, X_bio_14, X_bio_15, X_bio_18, X_bio_19 refers to mean diurnal range, isothermality, mean temperature of wettest quarter, mean temperature of driest quarter, precipitation of wettest month, precipitation of driest month, precipitation seasonality, precipitation of warmest quarter, precipitation of coldest quarter

Based on the VIFs between bioclimatic variables exceeding ten were excluded. There were 11 variables used in *Ae. albopictus* and *Ae. aegypti* prediction and 13 variables used in DENV prediction. According to the Jackknife test for DENV, the distribution of *Ae. albopictus* (82.7% contribution), *Ae. aegypti* (3% contribution) and the human population (11.8% contribution) are the main variables affecting DENV potential prevalence (Fig. 2). *Ae. albopictus* is more competent vector than *Ae. Aegypti.* As shown in Fig. 2, with the increase in the *Ae. aegypti* and *Ae. albopictus* density range, the probability of the occurrence of DENV rapidly increased.

**Fig. 3:**
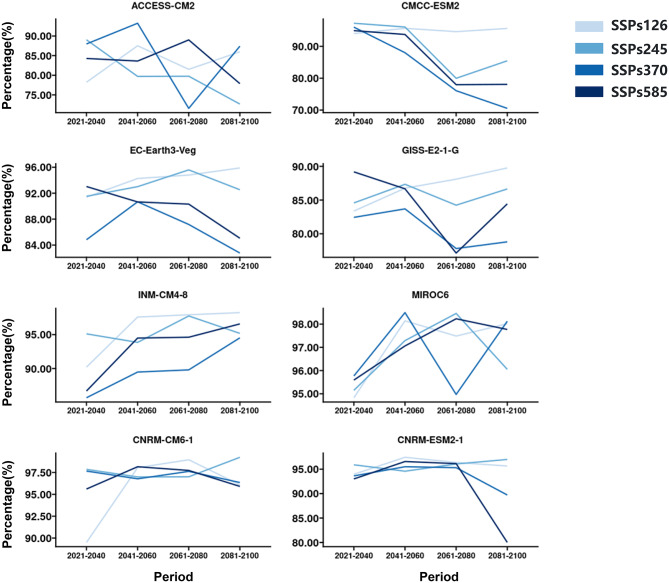
Potential total area for DENV under different SSPs (SSPs126, SSPs245, SSPs370, and SSPs585) of different climate scenarios in future periods (2021–2040, 2041–2060, 2061–2080, and 2081–2100) of the 21st century. The y-axis represents the percentage of the total area for DENV divided by the total area of Zhejiang Province of China

### Potential future risk of DENV in Zhejiang

**Fig. 4:**
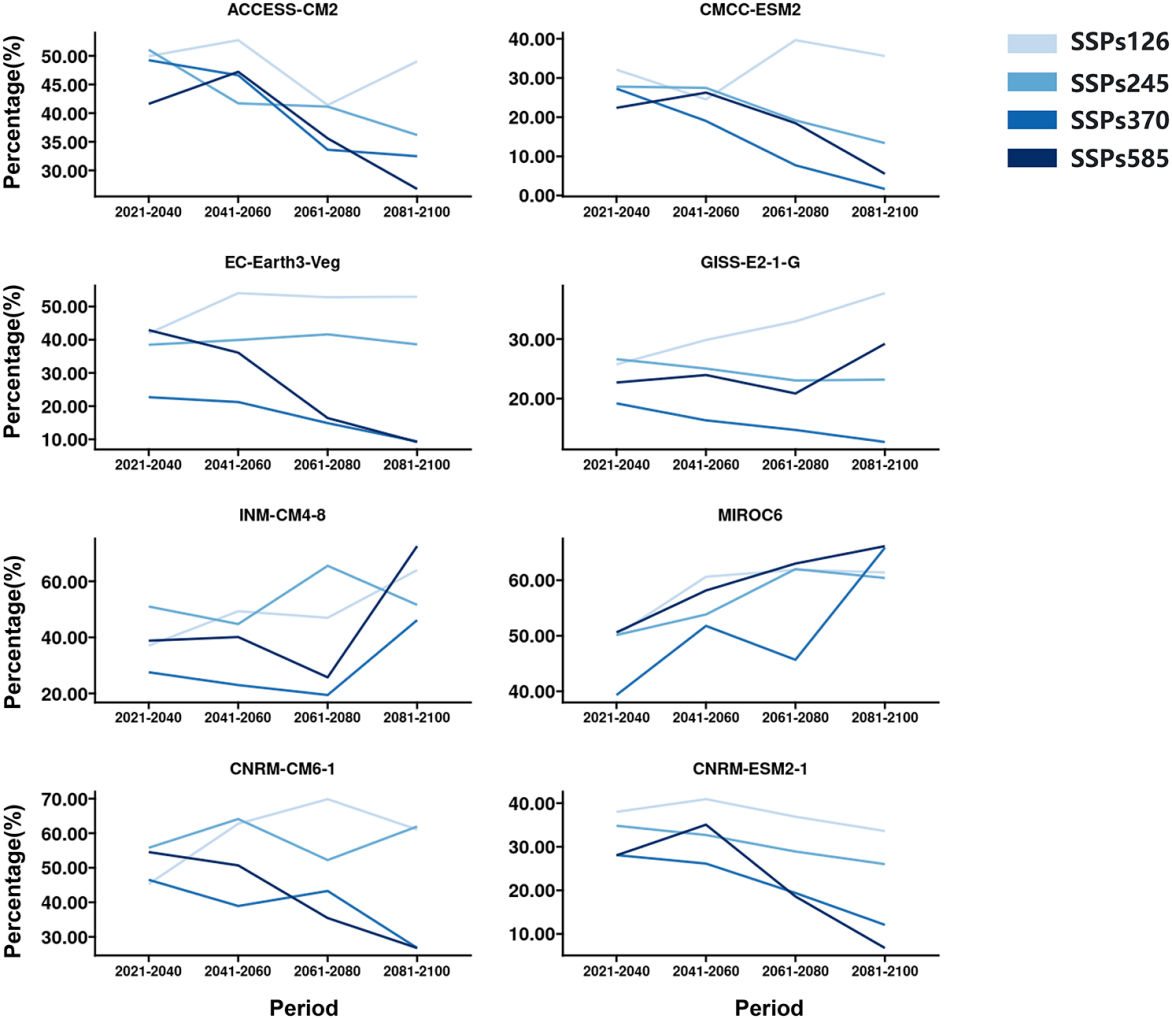
Potential high risk area for DENV under different SSPs (SSPs126, SSPs245, SSPs370, and SSPs585) of different climate scenarios in future periods (2021–2040, 2041–2060, 2061–2080, and 2081–2100) of the 21st century. The y-axis represents the percentage of high risk area for DENV divided by the total area of Zhejiang province of China

The potential distribution of DENV from 2021 to 2040, 2041–2060, 2061–2080, and 2081–2100 was predicted based on the previously constructed model. According to the results, if the development paths of society are sustainable and “green” pathways, during 1081–2100, the total risk (Fig. 3) area and the high risk (Fig. 4) will increase in the future 2081–2100 under SSP126 of EC-Earth3-Veg, GISS-E2-1-G, INM-CM4-8, MIROC6 and CNRM-CM-1. If the development path of society is Fossil-fueled development, during 2081–2100, the total risk (Fig. 3) area will decrease under SSP585 of ACCESS-CM2, CMCC-ESM2, EC-Earth3-Veg, GISS-E2-1-G and CNRM-ESM2-1, and the high risk (Fig. 4) area will decrease under SSP585 of ACCESS-CM2, CMCC-ESM2, EC-Earth3-Veg, CNRM-CM6-1 and CNRM-ESM2-1. The high risk (Fig. 4) area will first increase and then decrease under SSP585 of ACCESS-CM2, CMCC-ESM2 and CNRM-ESM2-1 in the future period of 2021–2100. The lowest total risk was predicted by SSP370 of CMCC-ESM2 during 2081–2100. The lowest high risk was predicted by SSP585 of CMCC-ESM2 during 2081–2100.

## Discussion

In this study, we aimed to predict the potential distribution of DENV in Zhejiang province under different SSPs. The ecological niche of primary vectors of DENV was first performed with historical climate data based on MaxEnt. Then, predicted results along with a set of bioclimatic variables were then included in MaxEnt model to analyze the risk region of DENV in Zhejiang province. Finally, the established model was utilized to predict spatial pattern of DENV risk in the different current and future scenarios in Zhejiang province.

MaxEnt is one of the most commonly used ecological niche models. Previous study showed that DENV outbreaks in China are significantly shaped by environmental factors, such as temperature, precipitation and land cover through MaxEnt model [38]. The MaxEnt model could correctly describe the breeding sites distribution of *Ae. Aegypti*, *Ae. albopictus* and DENV [39]. Variables with a variance inflation factor (VIF) of < 10 were left for model fitting to reduce collinearity, which adversely affects the accuracy and interpretability of the MaxEnt model [40]. Typically, AUC values above 0.9 indicate excellent model performance [41]. For MaxEnt model, the values of TSS is lower than the values of Area Under the Curve (AUC). This is often the case because TSS is a more stringent and less biased evaluation metric compared to AUC [37]. TSS is more sensitive and decreases as the model’s sensitivity and specificity decrease, along with an increase in omission errors and commission errors. The differences in AUC values between each model are smaller than the differences in TSS values, making TSS a more discriminative metric [42]. Although the use of AUC has been criticized [36, 37], it is still widely used for evaluating ecological niche models (ENMs). In addition to statistical considerations, we must also take into account the coherence with the biology and ecology of the species. In our study, the MaxEnt model was utilized, producing reliable results with an AUC value of 0.975 and a TSS value of 0.80 for predicting risk area.

Our findings indicated that *Ae. albopictus*, human population, *Ae. aegypti*, *Ae. albopictus* had significant impacts on DENV potential prevalence and made substantial contributions. Consistent with our findings, *Ae. albopictus* is more competent vector than *Ae. Aegypti.* Dengue outbreaks in China have primarily been attributed to *Ae. albopictus*, with *Ae. aegypti* being detected only in limited areas of southern and southwestern China in recent years [43–46]. Temporal fluctuations in *Ae. albopictus* density play a significant role in the local transmission of DENV. The monitoring results of *Ae. albopictus* in Hangzhou showed that July to August is the peak period of *Ae. albopictus* density [17], which could be may be the reason for the epidemic of DENV in Hangzhou in 2017. Additionally, in the process of urbanization, the increase of urban population density may lead to the prevalence of infectious diseases. Wiese et al. took neighborhood factors into account in addition to environmental variables using MaxEnt, and the combined model showed much better accuracy compared with the model with environmental variables exclusively [47]. As is reported, human density significantly influences dengue dynamics at fine spatial scales, such as city blocks and census tracts, with human population density acting as the primary driver [48]. The dense urban population and the presence of infected mosquitoes are crucial factors contributing to the transmission and prevalence of dengue fever. The implementation of strict prevention and control measures against COVID-19 by the Chinese government likely contributed to the containment of DENV transmission in Zhejiang, further supporting the notion these triggering factors for the DENV epidemic in the region.

Our results suggested that environmental factors may also play a role. The suitable temperature and precipitation range could promote the development and survival for *Ae. aegypti* and *Ae. albopictus.* Our findings indicated that temperatures during the driest quarter and precipitation of the wettest month also made contributions to DENV potential prevalence. Consistent with our study, rising temperatures during the driest quarter can enhance the survival rates of mosquitoes, consequently prolonging the duration of exposure to dengue hazards [49]. Similarly, researchers found that an increase in precipitation during the wettest month (over 8 mm) could create favorable comminity conditions for *Ades* mosquitoes [50]. Hence, our study emphasizes the need for vigilance against a potential DENV epidemic in light of ongoing increases in precipitation and temperatures.

The results revealed that the regions with a high potential for DENV epidemics were primarily located in Wenzhou, Jinhua, Quzhou, and Lishui, which are situated in the southern, western, west-eastern, and central parts of Zhejiang province. Our results align with the epidemiological characteristics of dengue fever in Zhejiang province from 2015 to 2019 [46]. During this period, Wenzhou and Jinhua experienced relatively high DENV risk and reported a larger number of DENV cases. Notably, Hangzhou witnessed a significant DENV outbreak, with 1,424 cases (1,201 indigenous and 223 imported) reported during 2017–2019. The occurrence of local cases in subsequent years may be attributed to imported cases from Southeast Asia and subsequent local transmission [51]. The extensive trade and collaboration with foreign regions or countries, especially in cities like Hangzhou, increase the risk of imported cases.

The growth and spread of DENV are intricately linked to social and economic development, global climate change, tourism, commerce, migrant workers, and accelerated urbanization. According to our findings, under SSP126, the risk for DENV in Zhejiang province is projected to increase in the future compared with the present. Even slight increases in temperature could provide substantial benefits to mosquito-borne disease, regardless of great efforts made by human societies to control carbon emissions. Aligned with our findings, in the best-case scenario SSP126 there will be a prolongation of the risk season for mosquito-borne disease in Brazil [52]. This highlights the need to integrate international climate protection policies with national disease preparedness efforts, including enhancing capacity for disease surveillance, diagnosis, and treatment. Additionally, under SSP585, a scenario characterised by Fossil-fueled development and limited greenhouse gas mitigation, the risk area for DENV in Zhejiang province is expected to initially expand and then decrease. This trend can be attributed to the rising temperatures in Zhejiang province, situated in the subtropical region, resulting from increased carbon dioxide emissions and global warming. Initially, the elevated temperatures create a more suitable environment for the survival and transmission of mosquito vectors. However, persistent overheating eventually reduces the vectors’ suitability for survival. Correspondingly, existing literature suggests that as temperatures rise, the regions most conducive to dengue risk shift to higher altitudes, while the tropical regions become unsuitable due to excessively high temperatures (over 35 °C) [53]. They suggest many cities in coastal eastern China and Japan are also likely to become suitable for dengue prevalence by 2050 [53]. Similar to our results, Xu also suggested a reduced risk region for Zika virus in the future SSP585 scenario, attributing it to environmental degradation and rising temperatures, which share the same vectors as DENV [54]. Our interpretation suggests that these results may be attributed to rising temperatures and changes in the vectors’ ecological adaptations. Our findings support the idea that effective vector control, community participation, health education, and environmental measures in high/moderate-risk regions could minimize the spatial diffusion and future pandemic potential of dengue fever in Zhejiang.

## Conclusion

Our study predicted that the current risk area in Zhejiang province of China, with the AUC value of 0.975 and the TSS value of 0.8, which indicated an accurate prediction. The future climate showed different changing trends according to the development paths of society. The implications of this research highlight the need for effective vector control measures, community engagement, health education, and environmental initiatives to mitigate the potential spread of dengue fever in high-risk regions of Zhejiang province.

## Electronic supplementary material

Below is the link to the electronic supplementary material.

Supplementary Material 1

## Data Availability

The datasets presented during the current study are available in the online repositories. The *Ae. albopictus* dataset can be accessed via https://www.gbif.org/species/1651430 [13 October 2022] and the *Ae. aegypti* dataset via https://www.gbif.org/species/1651891 [13 October 2022] on GBIF. For HealthMap, https://healthmap.org/, WorldClim, https://www.worldclim.org/, SEDAC, https://sedac.ciesin.columbia.edu/. The processed data are available on request from the corresponding author.
