# Supplementary material for: An ecological assessment of the potential pandemic threat of Dengue Virus in Zhejiang province of China
Source: BMC Infect Dis. 2023 Jul 17;23:473. doi: 10.1186/s12879-023-08444-0 (PMC10351185; doi:10.1186/s12879-023-08444-0)
Supplement: Supplementary file 1 — Supplementary Material 1 [file 12879_2023_8444_MOESM1_ESM.docx]

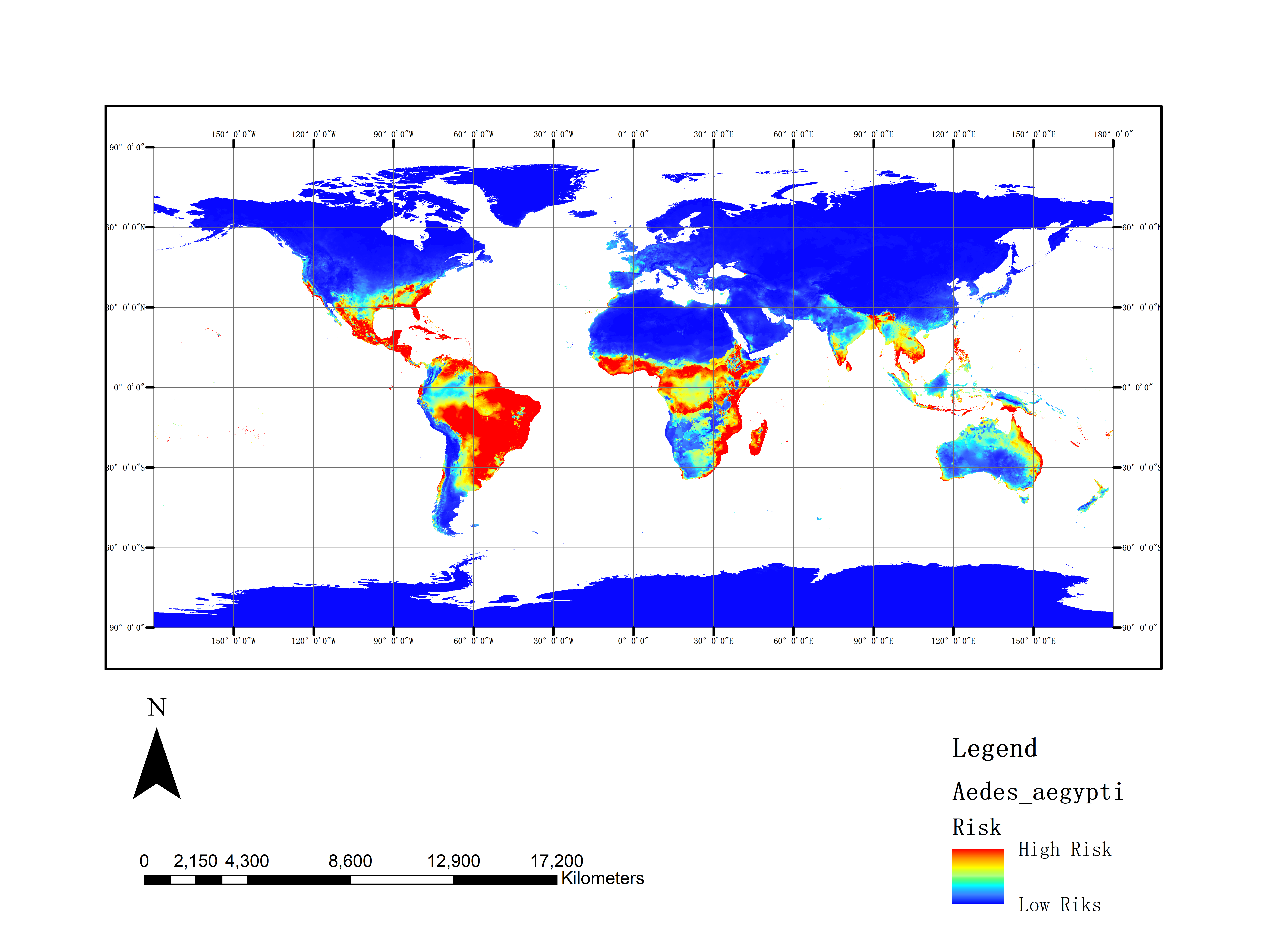


Figure S1 The rate of *Ae. aegypti* risk region of cities in the world.


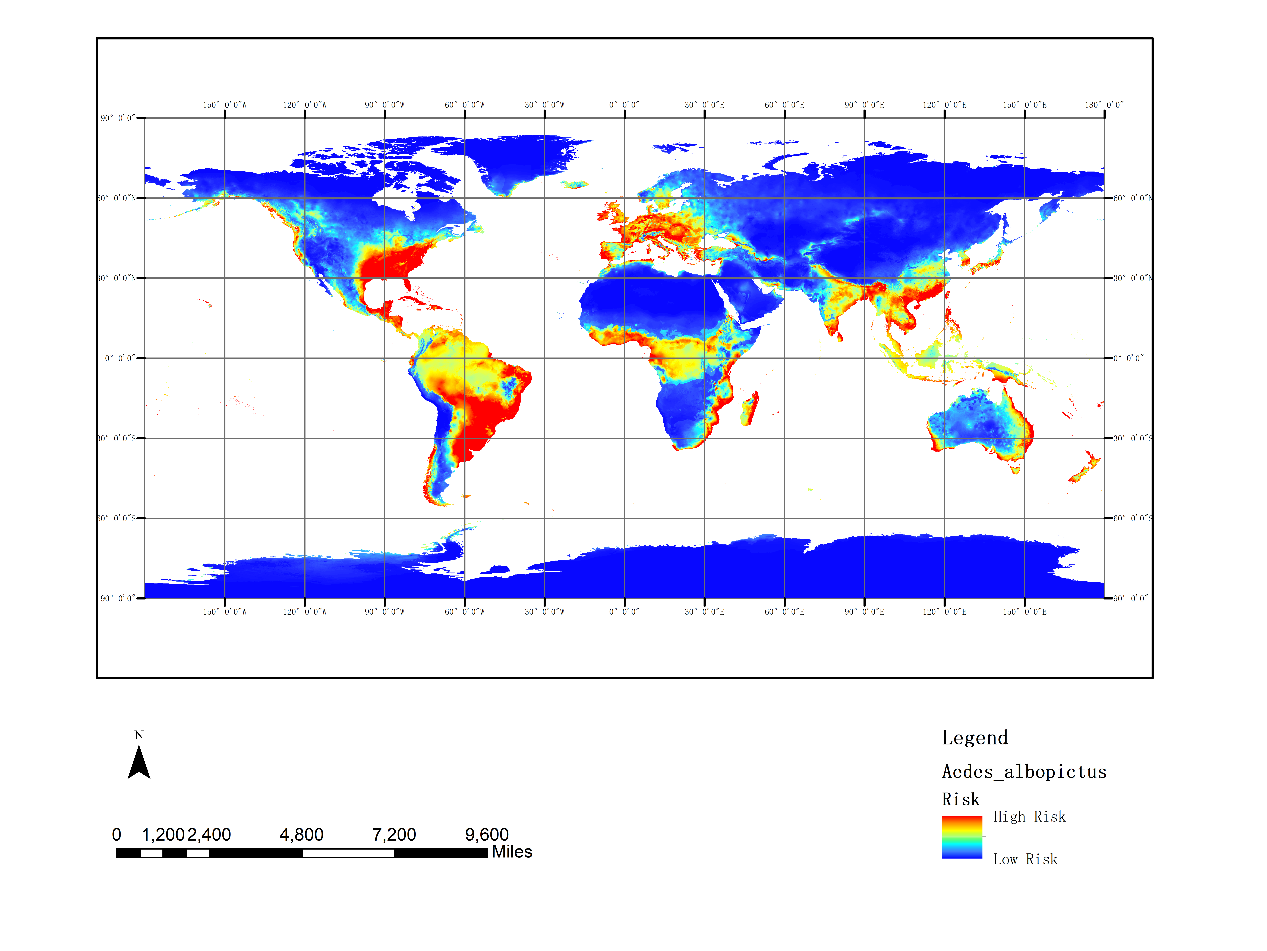


**Figure S2 The rate of *Ae. albopictus* risk region of cities in the world.**


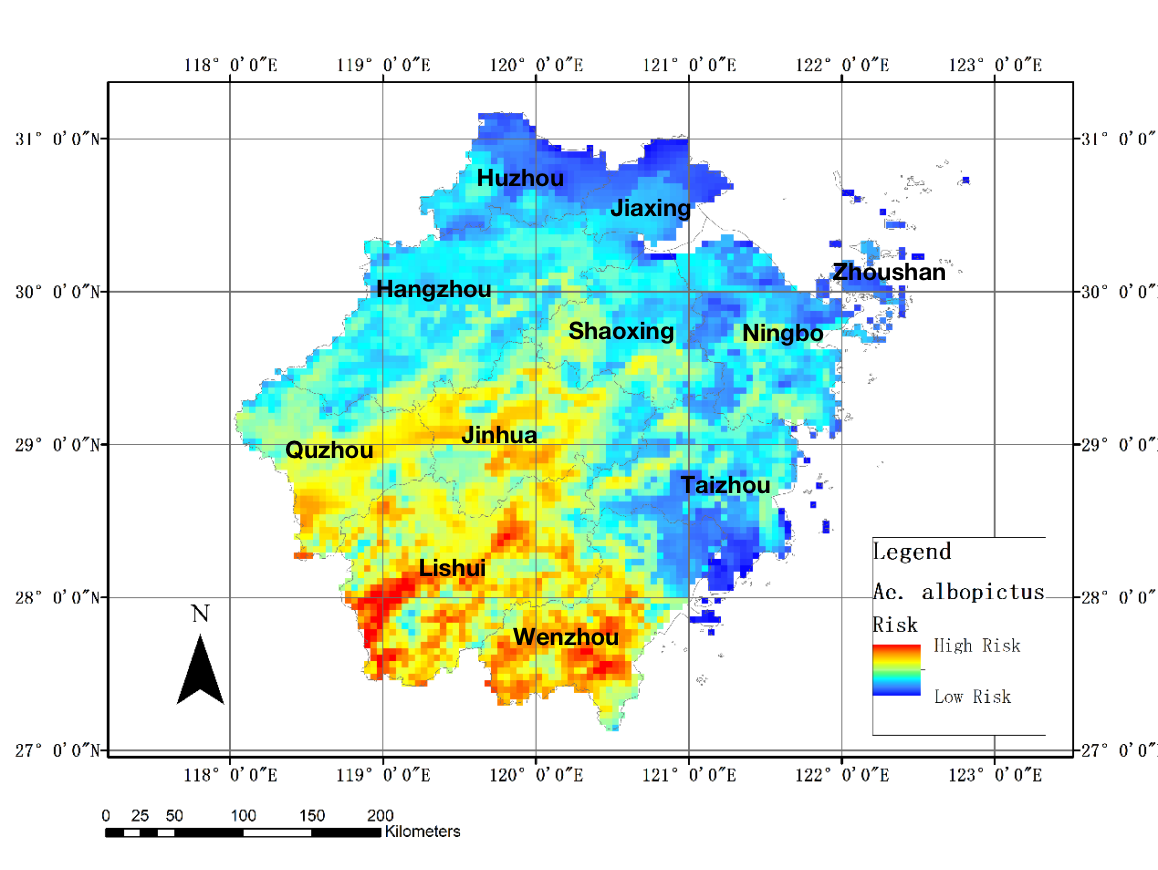


Figure S3 The rate of *Ae. albopictus* risk region of cities in Zhejiang Province.
